# Immunohistochemical Evaluation of Adaptor Protein FAM159B Expression in Normal and Neoplastic Human Tissues

**DOI:** 10.3390/ijms222212250

**Published:** 2021-11-12

**Authors:** Anna-Sophia Lieselott Beyer, Daniel Kaemmerer, Jörg Sänger, Katja Evert, Amelie Lupp

**Affiliations:** 1 Institute of Pharmacology and Toxicology, Jena University Hospital, 04474 Jena, Germany; anna-sophia.beyer@uni-jena.de; 2 Department of General and Visceral Surgery, Zentralklinik Bad Berka, 99438 Bad Berka, Germany; Daniel.Kaemmerer@zentralklinik.de; 3 Laboratory of Pathology and Cytology Bad Berka, 99438 Bad Berka, Germany; pathologie.bad-berka@t-online.de; 4 Department of Pathology, University of Regensburg, 93053 Regensburg, Germany; Katja.Evert@klinik.uni-regensburg.de; 5 Institute of Pathology, University Medicine of Greifswald, 17475 Greifswald, Germany

**Keywords:** FAM159B, adaptor protein, antibody, immunohistochemistry, neuroendocrine, tumour

## Abstract

FAM159B is a so-called adaptor protein. These proteins are essential components in numerous cell signalling pathways. However, little is known regarding FAM159B expression in normal and neoplastic human tissues. The commercially available rabbit polyclonal anti-human FAM159B antibody HPA011778 was initially characterised for its specificity using Western blot analyses and immunocytochemistry and then applied to a large series of formalin-fixed, paraffin-embedded normal and neoplastic human tissue samples. Confirmation of FAM159B’s predicted size and antibody specificity was achieved in BON-1 cells, a neuroendocrine tumour cell line endogenously expressing FAM159B, using targeted siRNA. Immunocytochemical experiments additionally revealed cytoplasmic expression of the adaptor protein. Immunohistochemical staining detected FAM159B expression in neuronal and neuroendocrine tissues such as the cortex, the trigeminal ganglia, dorsal root and intestinal ganglia, the pancreatic islets and the neuroendocrine cells of the bronchopulmonary and gastrointestinal tract, but also in the syncytiotrophoblasts of the placenta. FAM159B was also expressed in many of the 28 tumour entities investigated, with high levels in medullary and anaplastic thyroid carcinomas, parathyroid adenomas, lung and ovarian carcinomas, lymphomas and neuroendocrine tumours of different origins. The antibody HPA011778 can act as a useful tool for basic research and identifying FAM159B expression in tissue samples.

## 1. Introduction

Adaptor proteins are usually non-enzymes and are essential components that inherit important roles in various cell signalling pathways. In order to mediate protein–protein or protein–membrane interactions, they contain modular domains and/or linear peptide motifs. They are involved in regulating diverse aspects of cell surface receptor functions, including downstream signal transduction and transport or recycling of receptors. β-arrestin and its involvement in G-protein-coupled receptor signalling is a notable example of such an adaptor protein and its importance in the cell [1].

Another adaptor protein that is a Shisa-like protein is FAM159B. These Shisa-like proteins possess an N-terminal domain with six conserved cysteines and are thus related to the Shisa family of single transmembrane proteins that contain eight conserved cysteines. Vertebrates possess two forms of FAM159: FAM159A and FAM159B. FAM159B is thought to be a transmembrane adaptor that regulates other transmembrane proteins and receptors [1].

To identify genes with elevated expression in the human pancreas, Danielsson et al. [2] performed a genome-wide RNA sequencing analysis of the human transcriptome, followed by immunohistochemistry-based protein profiling to map proteins to their corresponding compartment(s) and cell type(s) within the pancreas. Among the identified upregulated proteins, several had not previously been described, including FAM159B, which was highly and specifically expressed in the islets and located at the cell membrane and in the cytoplasm. Distinct expression in neuroendocrine cells of the stomach mucosa was also observed, and FAM159B expression was consistent across all islet samples analysed from patients with type 1 or type 2 diabetes, as well as non-diabetic patients. The whole of the Langerhans islets showed FAM159B expression, leading to the conclusion that staining was probably not β-cell-specific. Therefore, FAM159B was proposed as a potential marker for total islet mass [2].

Using a simultaneous pancreatic single-cell, patch-clamp and RNA sequencing (patch–seq) approach to identify genes associated with islet cell function and dysfunction, Camunas-Soler et al. [3] found that FAM159B transcripts correlated positively with total exocytosis, early exocytosis, Ca^2+^ entry and Na^+^ currents of β-cells. Furthermore, after knockdown of *FAM159B* with a specific siRNA, a significant reduction of the exocytotic response was observed. In contrast to the findings of Danielsson et al. [2], FAM159B was thus proposed to be a β-cell-enriched molecule, a regulator of β-cell exocytosis and a marker of β-cell subsets. In another study, single-cell transcriptome profiling of human stem-cell-derived islet cells before and 6 months after transplantation into the kidney capsule of diabetic mice was performed, and the results were compared to data obtained from native cadaveric human islets [4]. Whereas FAM159B was expressed in native islets, its expression was not detectable in non-grafted β-cells, although distinct upregulation of its transcripts upon maturation after grafting was noted. These findings were further confirmed by immunohistochemical stains of in vitro-derived and transplanted stem cells, indicating FAM159B as a possible maturation marker of β-cells.

Beyond these limited data regarding the presence of FAM159B in adult islet cells and endocrine cells of the gut, little is known of FAM159B expression in other normal human tissues. Notably, no information is available related to the possible presence of FAM159B in human tumours and cancer cell lines.

Therefore, the aim of the present study was to evaluate the expression of FAM159B in a large series of formalin-fixed and paraffin-embedded human normal and neoplastic tissue samples using immunohistochemistry to obtain a broad expression profile of this protein in humans and elucidate its possible functions. For this purpose, the commercially available rabbit polyclonal anti-human FAM159B antibody HPA011778 (Atlas Antibodies, Bromma, Sweden) was initially characterised for its specificity using Western blot analyses and immuncytochemistry in the neuroendocrine tumour cell line BON-1, which endogenously expresses FAM159B. Our subsequent immunohistochemical investigations revealed high expression of FAM159B, particularly in pancreas islets, specific neuroendocrine cells of the intestinal tract and small-cell lung cancer (SCLC). Based on these findings, the range of neuroendocrine neoplasms studied was broadened and, ultimately, a diverse panel of bronchopulmonary and gastroenteropancreatic neoplasms (BP-NEN and GEP-NEN) of different malignancy and origin were examined for FAM159B expression. Staining results were then correlated with clinical data, such as tumour stage and grade, glucagon or insulin secretion and overall survival of the patients, and with the expression data of different neuroendocrine tumour markers.

## 2. Results

### 2.1. Characterisation of the Rabbit Polyclonal Anti-Human FAM159B Antibody HPA011778

**Figure 1:**
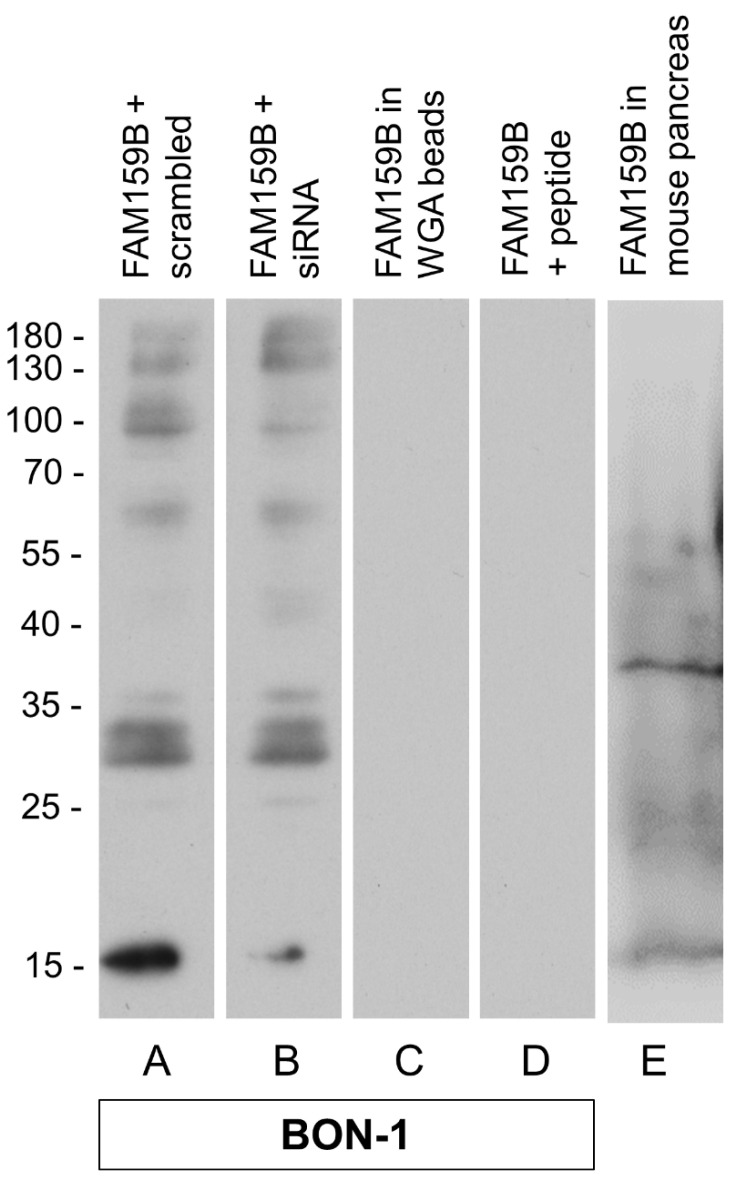
Specificity analysis of the polyclonal rabbit anti-human FAM159B antibody HPA011778. Western blot analysis of the supernatant from whole-cell preparations of BON-1 cells, which endogenously express FAM159B, after transfection with empty vector (scrambled) as a control (**A**) or specific siRNA (**B**). (**C**) Western blot analysis of wheat germ agarose (WGA) bead fractions containing glycosylated proteins. (**D**) Western blot analysis of the supernatant from whole-cell preparations when the antibody was pre-adsorbed with the immunising peptide. (**E**) Western Blot analysis of FAM159B in the cytosolic fraction of whole-organ preparations from mouse pancreas. Ordinate: migration of protein molecular weight markers (kDa). Representative results from one of three independent experiments are shown.

The specificity of HPA011778 was first tested using Western blot analyses. When the cytosolic fraction of endogenously FAM159B-expressing BON-1 cells was separated through electrophoresis and immunoblotted, the antibody recognised a band at approximately M_r_ = 16,000–18,000 (Figure 1A).

The observed molecular weight matched the expected weight of FAM159B, with M_r_ = 17,663 [5]. In contrast, no immunosignal could be detected in the wheat germ agarose (WGA) bead fraction, which contained the enriched glycosylated proteins (Figure 1C). After treatment of BON-1 cells with a targeted siRNA, the immunosignal in the cytosolic fraction was diminished (Figure 1B). To further confirm the specificity of the antibody, it was pre-adsorbed with its immunising peptide and then used for Western blot analysis, which revealed that the immunosignal was completely abolished (Figure 1D). As an endogenous control, immunoblots from the cytosolic fraction of whole-organ preparations from mouse pancreas were performed. Here, again, a band at approximately M_r_ = 16,000–18,000 was detected (Figure 1E).

**Figure 2:**
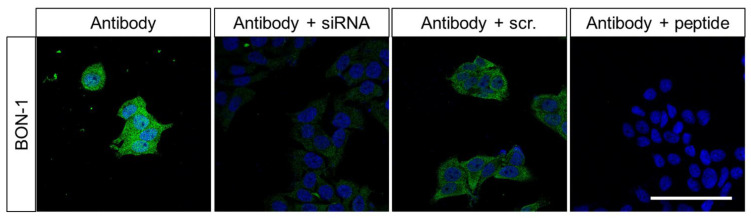
Immuncytochemical analysis of BON-1 cells endogenously expressing FAM159B. Cells remained untreated or were transfected with specific siRNA or an empty vector (scrambled) and then fixed and immunofluorescently stained with anti-FAM159B HPA011778. Right panel: staining using antibody HPA011778 pre-adsorbed with the immunising peptide. Green: immunosignal; blue: DAPI staining of DNA. All photomicrographs were captured at the same magnification. Scale bar: 100 µm.

For further evaluation of antibody specificity and characterisation of the subcellular localisation of the adaptor protein, we performed immunocytochemical analyses on BON-1 cells (Figure 2). The cells were left untreated or were transfected with a specific siRNA or an empty vector as control. They were then fixed and immunofluorescently stained with HPA011778. The untreated cells showed a bright immunosignal located throughout the cytosol. The signal remained the same with respect to its distribution pattern, but its intensity was reduced significantly in siRNA-transfected cells. BON-1 cells transfected as a control with an empty vector showed a similar immunosignal distribution and intensity to untreated cells. After pre-adsorption of the antibody with the peptide used for rabbit immunisations, complete repression of the immunosignal was again observed.

### 2.2. Immunohistochemical Detection of FAM159B Expression in Normal Human Tissues

**Figure 3:**
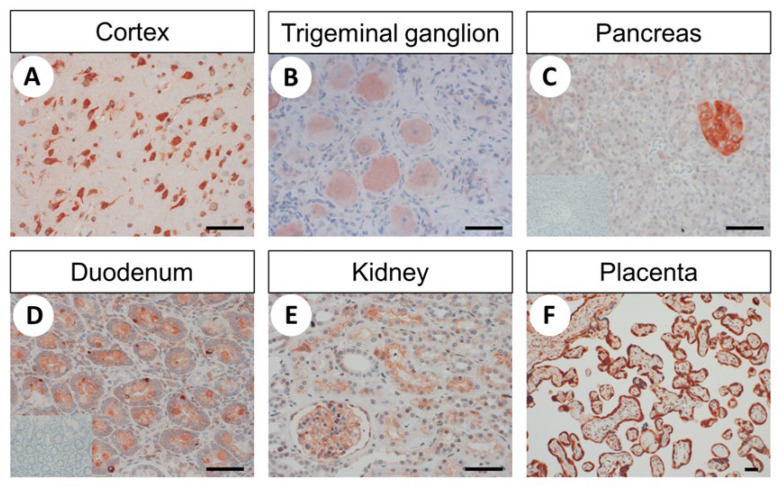
Immunohistochemical detection of FAM159B expression in different non-neoplastic human tissues. Immunohistochemical staining (red–brown colour), counterstaining with haematoxylin. Scale bar: 100 µm (**A**–**E** and 20 µm (**F**). Insets in (**C**) and (**D**): for adsorption controls, the anti-FAM antibody HPA011778 was incubated with 10 µg/mL of the immunising peptide.

The rabbit polyclonal anti-FAM159B antibody HPA011778 was then applied to immunohistochemical stainings of different human non-neoplastic tissue samples. For most samples, immunostaining was localised to the cytoplasm of the cells, but in some cases a staining of the plasma membrane was also observed. A selection of FAM159B-positive normal and neoplastic human tissue samples was incubated with HPA011778 pre-adsorbed with its immunising peptide, which in all cases caused a complete loss of the immunosignal (see insets in Figure 3 and Figure 7).

A distinct staining was detected in the pyramidal cells of the human cortex, trigeminal ganglia, dorsal root and intestinal ganglia, pancreatic islets, endocrine cells of the bronchopulmonary and intestinal tract and syncytiotrophoblasts of the placenta (Figure 3). In addition, slight immunostaining was noticed in bronchial and intestinal epithelia, in the epithelium of the larger bile ducts in the liver and in that of the gallbladder. Within human kidneys, the adaptor protein was found in mesangial cells of the glomeruli, the visceral and parietal layer of Bowman’s capsule and the distal tubules (Figure 3E). No expression of FAM159B was observed for the proximal tubules, loops of Henle, collecting ducts or blood vessels. Furthermore, no noticeable immunosignals could be detected in lung, heart, thymus, spleen, lymph node or liver tissue.

**Figure 4:**
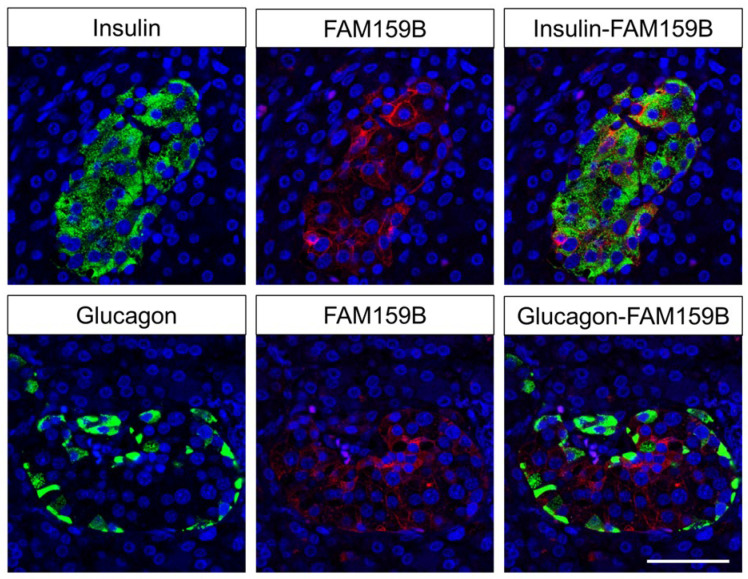
Double-labelling immunofluorescence analysis of human pancreatic islets. Sections were dewaxed and microwaved in citric acid. Adjacent tissue sections were incubated with rabbit polyclonal anti-FAM159B antibody HPA011778 together with mouse monoclonal antibodies against insulin or glucagon. Labelling for FAM159B was visualised using a Cy3-conjugated anti-rabbit antibody (red) and for insulin or glucagon using an Alexa-Fluor-488-conjugated anti-mouse antibody (green). Scale bar: 100 µm.

To examine FAM159B expression in the pancreatic islets in more detail, we performed additional double-labelling fluorescence experiments for glucagon and insulin, which confirmed the presence of FAM159B in insulin-producing β-cells and, to some extent, in glucagon-releasing α-cells (Figure 4).

**Figure 5:**
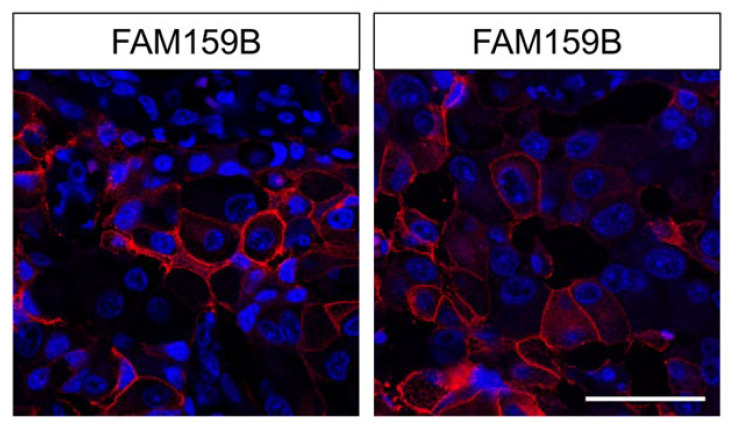
Immunofluorescence analysis of human exocrine pancreas. Sections were dewaxed and microwaved in citric acid. Tissue sections were incubated with rabbit polyclonal anti-FAM159B antibody HPA011778, followed by Cy3-conjugated anti-rabbit antibody (red). Scale bar: 100 µm.

Fluorescent staining of the pancreas for FAM159B additionally revealed membranous expression in the acinar cells of the exocrine pancreas (Figure 5), which was not evident when the samples were evaluated with light microscopy.

### 2.3. Immunohistochemical Detection of FAM159B Expression in Different Human Tumours and Tumour Cell Lines

**Figure 6:**
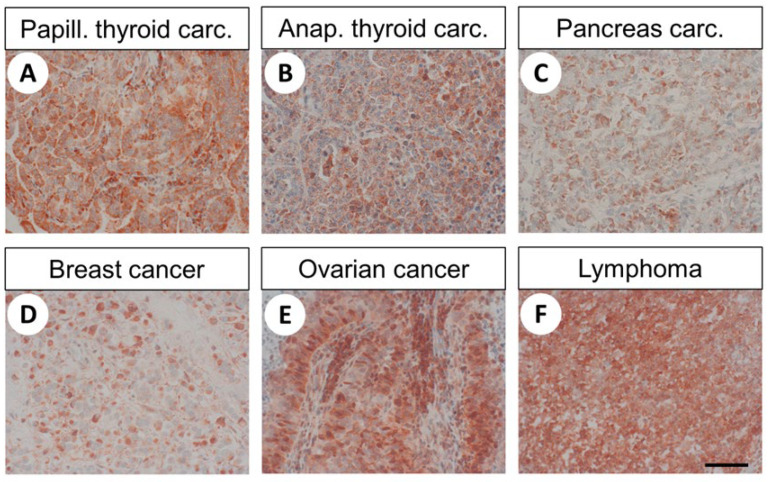
Immunohistochemical detection of FAM159B expression in different human tumour entities. Immunohistochemical staining (red–brown colour), counterstaining with haematoxylin. Scale bar: 100 µm (**A**–**F**).

The distribution pattern of FAM159B in the different human tumour samples investigated is summarised in Table 1, while Figure 6 shows representative results of these immunostaining experiments.

Again, both cytoplasmic and membranous staining patterns were observed. Furthermore, considerable inter- and intra-individual variability in FAM159B expression was noticed. For several samples, only very small sections of the tumour showed a strong staining signal, while the remainder lacked any expression, resulting in an overall low immunoreactivity score (IRS) value. Overall, moderate-to-strong FAM159B expression (average IRS > 5) was seen in pituitary adenomas, medullary and anaplastic thyroid carcinomas, parathyroid adenomas, adenocarcinomas of the lung, SCLCs, ovarian carcinomas and lymphomas. Low expression of FAM159B (3 ≤ IRS ≤ 5) occurred in papillary thyroid carcinomas, squamous cell carcinomas of the lung, gastric adenocarcinomas, colon carcinomas, pancreatic adenocarcinomas, breast carcinomas, cervical cancer and melanomas. No or very weak expression (IRS < 3) was found in glioblastomas, follicular thyroid carcinomas, gastrointestinal stromal tumours, hepatocellular and cholangiocellular carcinomas, renal clear cell cancer, urinary bladder cancer, pheochromocytomas, endometrial cancer, prostate and testicular cancer and sarcomas (Table 1).

With respect to FAM159B expression in various tumour cell lines, a very strong immunosignal was obtained in the neuroendocrine tumour cell line BON-1, SCLC cell line OH-1 and prostate cancer cell lines PC-3, DU145 and LNCaP. A moderate-to-strong FAM159B expression was observed in neuroblastoma cell lines SK-N-SH and SH-SY5Y, SCLC cell lines NCI-h69 and NCI-h82, hepatocellular carcinoma cell lines SK-HEP-1 and HuH-7, colon carcinoma cell lines SW480, HT-29 and LoVo, renal cell carcinoma cell line A498, urinary bladder carcinoma cell line RT-112, breast cancer cell lines MCF-7 and BT-474, ovarian carcinoma cell line A2780 and cervical cancer cell lines SW756, SiHa, CaSki and HeLa. A very weak expression was observed in the lung adenocarcinoma cell line A549, hepatocellular carcinoma cell lines HepG2 and Hep3B, ovarian carcinoma cell line SK-OV-3, urinary bladder carcinoma cell line T24, cervical cancer cell line ME-180 and the epidermoid carcinoma cell line A431. Neuroblastoma cell line SiMa and the breast cancer cell line MDA-MB-231 were devoid of FAM159B immunosignal.

### 2.4. FAM159B Expression in Bronchopulmonary and Gastroenteropancreatic Neuroendocrine Neoplasms

#### 2.4.1. Patient Characteristics

The evaluated neuroendocrine neoplasms originated from 144 (51.8%) male and 125 (45.0%) female patients; for 9 (3.2%) patients, the sex was unknown. The overall mean age of the patients at diagnosis was 58.5 years (median: 59.7 years; range: 12.1–83.9 years). Forty-seven (16.9%) of the corresponding primary tumours were classified as T1, 46 (16.6%) as T2, 57 (20.5%) as T3 and 24 (8.6%) as T4. In 104 (37.4%) cases, the extent of the primary tumour was unknown. One hundred and twenty-three (44.2%) patients already had lymph node metastases at diagnosis, 83 (29.9%) patients showed none, and the lymph node status was unknown for 72 (25.9%) patients. One hundred and ten (39.5%) patients had distant metastases, 89 (32.0%) had none, and no data were available for 79 (24.4%) patients. Of the 172 patients with GEP-NEN, 17 patients (9.9%) had UICC stage I disease, 12 (7.0%) had stage II disease, 23 (13.4%) had stage III disease, 98 (57.0%) had stage IV disease, and records of 22 (12.7%) patients had no information regarding UICC stage. The stage of the disease was not determined for the 97 bronchopulmonary tumour patients and the 9 patients with unknown tumour origin. Regarding histological grading, 99 (35.6%) patients had grade 1, 94 (33.8%) patients had grade 2 and 76 (27.3%) patients had grade 3 tumours. Tumour grading was unknown for 9 (3.2%) patients.

The median follow-up time was 59.7 months overall. One hundred and fifty-eight (56.8%) patients were alive at the end of the follow-up period, 87 (31.3%) had died due to tumour-related causes and 33 (11.9%) had no data available. The median survival time of patients who died during follow-up was 27.7 months, differing significantly by sex (25 months for males vs. 43.8 months for females).

#### 2.4.2. FAM159B Expression Pattern

**Figure 7:**
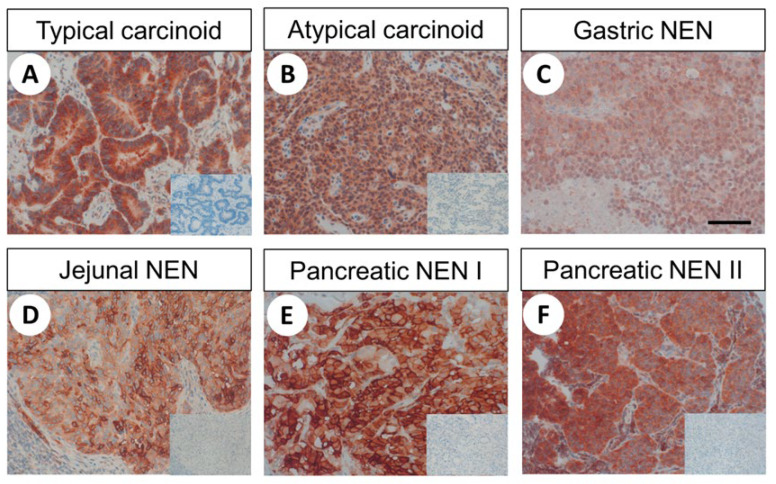
FAM159B expression pattern in different bronchopulmonary and gastroenteropancreatic neuroendocrine tumour entities. Immunohistochemical staining (red–brown colour), counterstaining with haematoxylin. Scale bar: 100 µm (**A**–**F**. Insets in (**A**,**B**,**D**,**E**,**F**): for adsorption controls, the anti-FAM antibody HPA011778 was incubated with 10 µg/mL of the immunising peptide.

Representative stainings of different BP-NEN and GEP-NEN are shown in Figure 7.

**Figure 8:**
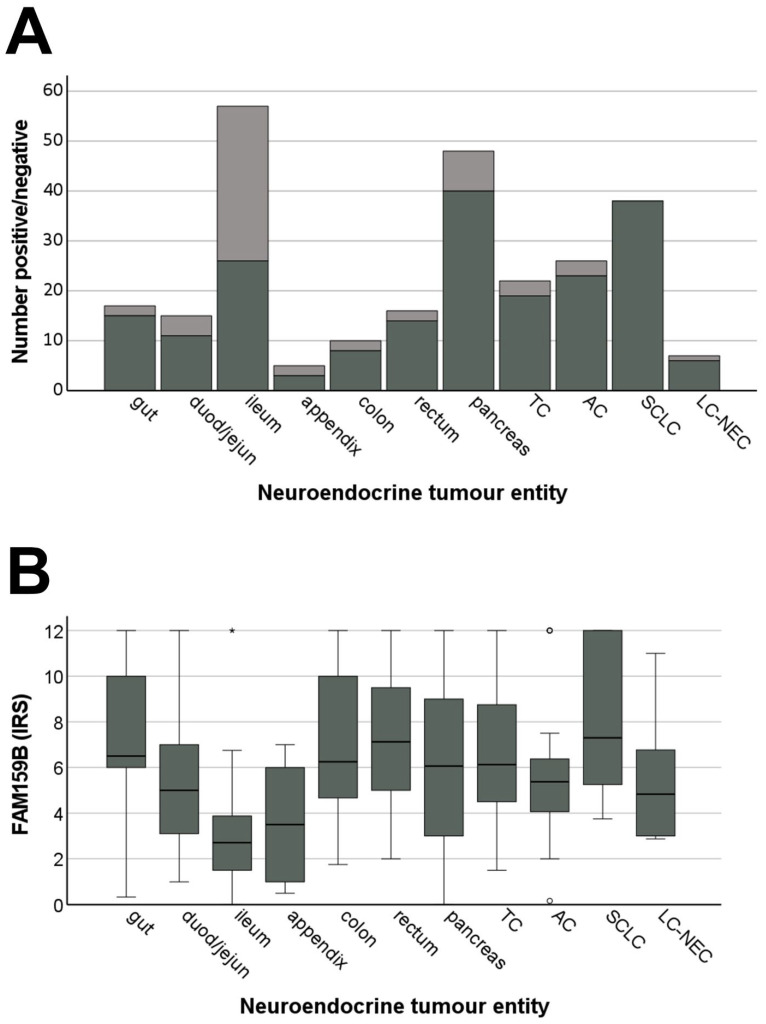
Expression profile of FAM159B in different bronchopulmonary and gastroenteropancreatic neuroendocrine tumour entities. (**A**) Number of FAM159B-positive (black) and FAM159B-negative (grey) cases within the different neuroendocrine tumour entities. Tumours were only considered positive at immunoreactivity score (IRS) values ≥ 3. (**B**) FAM159B expression levels (IRS values) in different neuroendocrine tumour entities. Depicted are median values, upper and lower quartiles and minimum and maximum values, as well as outliers. Outliers were defined as follows: circles: mild outliers (data points between 1.5 and 3 times above the upper quartile or below the lower quartile); asterisks: extreme outliers (data more than 3 times above the upper or below the lower quartile). TC: typical carcinoid of the lung; AC: atypical carcinoid of the lung; SCLC: small-cell lung cancer; LC-NEC: large-cell neuroendocrine carcinoma; gut: gastroenteropancreatic neuroendocrine tumours (GEP-NEN) from the gut; duod/jejun: GEP-NEN from the duodenum or jejunum; ileum: GEP-NEN from the ileum; colon: GEP-NEN from the colon; rectum: GEP-NEN from the rectum; pancreas: pancreatic neuroendocrine tumour.

Again, both cytoplasmic and membranous expression was observed, and substantial variation in immunostaining across individual patients and sometimes even between different samples originating from the same patient was noticed. These findings are illustrated by the lengths of the boxes and whiskers in Figure 8b. For pancreatic tumours, for example, the IRS values ranged from 0 (no expression) to 12 (maximum expression). Regarding the percentage of FAM159B-positive tumours (IRS ≥ 3) (Figure 8a) and the extent of expression (Figure 8b), the protein was most strongly expressed in SCLC (100%; median IRS: 7.5), followed by neuroendocrine tumours from the gut (88.2%; median IRS: 6.5), colon (81.8%; median IRS: 6.0), rectum (86.7%; median IRS: 7.0) and pancreas (83.3%; median IRS: 6.1), as well as in TC (86.4%; median IRS: 6.1). Of all GEP-NEN tested, tumours located in the ileum showed the lowest FAM159B expression (46.6%; median IRS: 3).

#### 2.4.3. Correlations with Clinical Data

Analysing the collective data of all BP-NEN and GEP-NEN revealed a significant difference between the FAM159B IRS scores for patients with or without lymph node metastases at diagnosis. Patients without lymph node metastases showed higher FAM159B IRS values (mean ± SEM: 5.817 ± 0.366 and 4.797 ± 0.280, respectively; Mann–Whitney *U* test: *p* = 0.035). In contrast, a significant positive correlation was observed between FAM159B IRS values and tumour grade (r_sp_ = 0.204; *p* < 0.001) and levels of the proliferation marker Ki-67 (r_sp_ = 0.306; *p* < 0.001). Nonetheless, Kaplan–Meier analysis revealed that FAM159B expression does not influence patient overall survival. No difference between FAM159B-positive or -negative tumours was observed when using IRS values of 3 (threshold of positivity) or 6 (close to the overall median IRS value of 5.7) as the cut-off value. When analysing interrelationships with several typical markers of, and receptors for, neuroendocrine tumours determined on the same set of samples as part of previous studies [6,7,8], a correlation was found between the IRS of FAM159B and the IRS values of the dopamine receptor D2, somatostatin receptors (SST)1, 3, 4 and 5, chemokine receptor CXCR4 and programmed death ligand 1 (PD-L1) (Table 2).

If only the BP-NEN were considered, no correlation between the IRS values of FAM159B and the presence of lymph node metastases was seen, although the positive correlation between FAM159B expression with tumour grade (r_sp_ = 0.139; *p* = 0.004) and Ki-67 index (r_sp_ = 0.218; *p* < 0.001) persisted. Again, Kaplan–Meier analysis could not demonstrate any statistically significant differences between patients with FAM159B-positive or -negative tumours. However, a positive correlation between IRS values of FAM159B and those of SST4 and CXCR4 was noted, as well as a trending positive association with PD-L1 (Table 2).

Analyses of only GEP-NEN revealed significantly higher FAM159B IRS values in patients free of lymph node metastases vs. those with metastases (mean ± SEM: 5.979 ± 0.624 vs. 4.431 ± 0.296, respectively; Mann-Whitney *U* test: *p* = 0.041). As previously observed, FAM159B IRS was significantly positively correlated with tumour grade (r_sp_ = 0.198; *p* < 0.001) and Ki-67 levels (r_sp_ = 0.279; *p* < 0.001). Similarly, Kaplan–Meier analysis showed no influence of FAM159B expression on overall survival of patients. When evaluating the associations between FAM159B and typical neuroendocrine tumour markers, positive associations were observed between the IRS values of FAM15B and those of the dopamine receptor D2, SST1, SST2, SST3, SST5, CXCR4 and PD-L1.

Finally, because double-labelling experiments have shown the presence of FAM159B in insulin-producing β-cells and in some glucagon-producing α-cells, pancreatic neuroendocrine tumours were additionally evaluated for insulin or glucagon expression. Here, a significant relationship was found between the IRS values of FAM159B and those of insulin (r_sp_ = 0.236; *p* = 0.016) but not of glucagon (r_sp_ = 0.162; *p* = 0.096). Furthermore, a positive correlation was observed between the IRS values of FAM159B and neuron-specific enolase (NSE) (r_sp_ = 0.217, *p* = 0.035) but not chromogranin A (r_sp_ = −0.029; *p* = 0.785).

## 3. Discussion

### 3.1. Characterisation of the Rabbit Polyclonal Anti-Human FAM159B Antibody HPA011778

#### 3.1.1. Western Blot Analysis

The specificity of HPA011778 was demonstrated in BON-1 cells that endogenously express FAM159B using Western blot analysis. The antibody selectively detected the adaptor protein in the supernatant (cytoplasmic fraction) of prepared cell lysates. As no signal was detected when analysing the WGA bead fraction, FAM159B seems to lack noticeable glycosylation within BON-1 cells. The specificity of the antibody for FAM159B was further demonstrated by transfection of BON-1 cells with a specific siRNA and by use of pre-adsorption controls with the immunising peptide. We also confirmed the adaptor protein’s previously predicted size of approximately 17 kDa both in BON-1 cells and in mouse pancreas.

#### 3.1.2. Immunocytochemistry

We applied immunocytochemistry to characterise FAM159B subcellular localisation. Endogenously expressed FAM159B in BON-1 cells is found within the cytosol, most likely within distinct vesicles or associated with other proteins. The FAM159B immunosignal was significantly diminished after BON-1 cells were transfected with a specific siRNA. The reduction in signal intensity represents successful transfection and thus decreased protein synthesis. Because the cells were incubated for 24 h after the start of transfection, FAM159B seems to have a lifespan of approximately 24 h, as the signal intensity would not be reduced if the lifespan was much longer. No signal would have been detected at this time point if FAM159B’s lifespan was much shorter than 24 h.

#### 3.1.3. Immunohistochemistry

In formalin-fixed, paraffin-embedded human tissue samples, the polyclonal anti-FAM159B antibody yielded a highly efficient and selective immunohistochemical staining of distinct cell populations. Through positive staining of pancreatic islets and endocrine cells of the gastrointestinal tract, the only existing immunohistochemical data of FAM159B expression could be confirmed [2,4]. As another proof of specificity, the immunosignals were completely abolished after pre-absorption of the antibody with its immunising peptide.

### 3.2. FAM159B Expression in Normal Human Tissues

In the present study, for the first time, FAM159B expression has been evaluated and visualised in various normal human tissues using immunohistochemistry and double-labelling experiments. A strong expression of FAM159B was found in particular in neuronal structures such as pyramidal cells of the cortex, the trigeminal ganglia, dorsal root and enteric ganglia and neuroendocrine tissues and cells, including pancreatic islets and neuroendocrine cells of the bronchopulmonary and gastrointestinal tracts, but also in syncytiotrophoblasts of the placenta. Because all these tissues and cells are involved in the release of various neurotransmitters and hormones, FAM159B may directly or indirectly play a role in their secretory function. In addition to neuronal and neuroendocrine cells, FAM159B was found to be expressed in various epithelia, such as bronchial, gastrointestinal, bile duct and gallbladder epithelia and in epithelia of the distal tubules of the kidney. At these sites, FAM159B may be involved in ion transport processes. The presence of FAM159B in neuroendocrine cells of the intestinal tract and in pancreatic islets has been shown previously [2,3,4] and has been demonstrated to be associated with exocytosis and with Ca^2+^ and Na^+^ ion currents [3]. However, the literature disagrees regarding which cells of the pancreatic islets express the protein. Whereas Danielsson et al. [2] proposed FAM159B as a potential marker for total islet mass, Camunas-Soler et al. [3] concluded from their studies that FAM159B is solely a marker of β-cell subsets. In our investigation, the adaptor protein showed homogenous expression throughout the whole of the islets under light microscopy. Subsequent immunofluorescence double-labelling experiments, however, revealed FAM159B to be clearly present in insulin but only to some extent in glucagon-producing islet cells. The assumption of a predominant presence of FAM159B in insulin-secreting cells is further corroborated by a tendency towards positive correlation between expression of FAM159B and insulin, but not glucagon, in pancreatic neuroendocrine tumours.

### 3.3. FAM159B Expression in Human Neoplastic Tissues

In the present study, a wide range of different human tumour entities and cancer cell lines was evaluated for possible expression of FAM159B. To date, no respective data are available from the literature. Our immunohistochemical studies revealed strong expression of FAM159B in tumours of endocrine or neuroendocrine origin, such as pituitary adenomas, BP-NEN and GEP-NEN, medullary and anaplastic thyroid carcinomas and parathyroid adenomas. This finding may be due to the fact that moderate-to-strong expression of FAM159B is normally observed in the corresponding non-neoplastic endocrine and neuroendocrine cells. However, intense FAM159B expression was not confined to tumours of endocrine or neuroendocrine origin. A moderate-to-strong presence of the protein was also found, e.g., in lung adenocarcinomas, ovarian carcinomas and lymphomas. Additionally, in many other tumour entities (despite overall low IRS values), individual tumours showed high FAM159B expression. In contrast, pheochromocytomas, neuroendocrine tumours originating from the adrenal medulla, did not noticeably express the adaptor protein. Fittingly, cancer cell lines with or without a neuronal or neuroendocrine background showed similarly strong immunosignals. Additionally, in BP-NEN and GEP-NEN, distinct differences in FAM159B expression were observed depending on the origin of the primary tumour. Together, these findings suggest functions of FAM159B that go beyond direct or indirect participation in neurotransmission and/or hormone secretion. Correlations with different markers for neuroendocrine tumours revealed a positive relationship between FAM159B and NSE (but not with chromogranin A), as well as with various receptors and membrane proteins commonly expressed in these tumours (but also in others), associated either with good prognosis, such as the D2 dopamine receptor and the different somatostatin receptors or with a negative prognosis, such as the chemokine receptor CXCR4 and PD-L1 [7,9]. Therefore, it is not surprising that no association between FAM159B expression and patient outcomes was seen. This finding may also explain the contradictory results of a positive association between FAM159B expression and tumour proliferation rate or grade and higher FAM159B levels in patients without lymph node metastases.

## 4. Materials and Methods

### 4.1. Antibody

The rabbit polyclonal anti-FAM159B antibody (HPA011778) was purchased from Atlas Antibodies (Bromma, Sweden). The sequence of the peptide used for the immunisations of the rabbits was as follows: TKPQRLDTGLKLQHLEASSTQEGKSNGKTKALNSNAASNATN ETYYEADDIIQEKTMDATQIHIA. The respective peptide PrEST Antigen FAM159B (APrEST71583) was also obtained from Atlas Antibodies (Bromma, Sweden).

### 4.2. Western Blot Analysis

BON-1 cells (DMSZ, Braunschweig, Germany), a neuroendocrine tumour cell line endogenously expressing FAM159B, were seeded into 60 mm Petri dishes and grown to 80% confluence. Cells were lysed in detergent buffer (150 mM NaCl, 50 mM Tris-HCl (pH 7.4), 5 mM EDTA, 1% Triton X-100, 0.5% Na-deoxycholate, 0.1% SDS, 100 mM phenylmethylsulfonylfluoride, 10 mg/mL leupeptin, 5 mg/mL aprotinin, 1 mg/mL pepstatin A). For detection of FAM159B in mouse pancreas, 50 mg of tissue was weighed out, 200 µL of the detergent buffer was added and the pancreas was sonicated for 10 s. Afterwards, the samples were gently inverted for 1 h at 4 °C before centrifugation for 30 min at 14,800 g at 4 °C. All samples were then treated with wheat germ lectin–agarose (WGA) beads. The supernatant of the samples, as well as the bead fractions, were subjected to 10% SDS-polyacrylamide gel electrophoresis and immunoblotted onto polyvinylidene fluoride membranes. The blots were then incubated with rabbit polyclonal anti-FAM159B antibody HPA011778 at a dilution of 1:500 overnight at 4 °C, followed by incubation with a peroxidase-conjugated secondary anti-rabbit antibody (dilution 1:5000; Santa Cruz Biotechnology, Dallas, TX, USA) and enhanced chemiluminescence detection (Amersham, Braunschweig, Germany).

When indicated, the expression of endogenous FAM159B in BON-1 cells was silenced using a chemically synthesised, single-stranded RNA oligonucleotide (siRNA; ID 264018; Ambion by Life Technologies (Thermo Fisher Scientific), Waltham, MA, USA) according to the manufacturer’s instructions. An empty vector was used as a negative control (Santa Cruz Biotechnology, Dallas, TX, USA).

For adsorption controls, HPA011778 was pre-incubated for 2 h at room temperature with 10 µg/mL of the peptide PrEST antigen (Atlas Antibodies, Bromma, Sweden) used for rabbit immunisations.

### 4.3. Immuncytochemistry

BON-1 cells were grown overnight on glass coverslips until 80% confluence was achieved. After washing of the cells with phosphate-buffered saline (PBS), cells were fixed with 4% paraformaldehyde and 0.2% picric acid in phosphate buffer (pH 6.9) for 20 min at room temperature. After thorough washing with PBS, cells were incubated with anti-FAM159B HPA011778 overnight at 4 °C, followed by incubation with an Alexa-Fluor-488-conjugated secondary antibody (Invitrogen, Karlsruhe, Germany; dilution 1:5000) for 2 h at room temperature. Samples were then mounted (Invitrogen Fluoromount-G, with DAPI; Thermo Fisher Scientific, Waltham, MA, USA) and examined using a Zeiss LSM 510 META laser scanning confocal microscope (Jena, Germany).

If required, endogenous FAM159B expression in BON-1 cells was silenced using a chemically synthesised, single-stranded RNA oligonucleotide (siRNA; ID 264018; Ambion by Life Technologies (Thermo Fisher Scientific), Waltham, MA, USA) according to the manufacturer’s instructions. An empty vector was used as a negative control (Santa Cruz Biotechnology, Dallas, TX, USA).

When indicated, HPA011778 was pre-adsorbed for 2 h at room temperature with 10 µg/mL of the peptide PrEST antigen (Atlas Antibodies, Bromma, Sweden) used for rabbit immunisations.

### 4.4. Immunohistochemical Evaluation of FAM159B Expression on Normal and Neoplastic Human Tissues and Human Tumour Cell Lines

#### 4.4.1. Tumour Specimens

For the initial assessment of FAM159B expression in various human tumour specimens, 259 formalin-fixed and paraffin-embedded tumour samples (Table 1) were obtained from the Department of Pathology of the Ernst Moritz Arndt University (Greifswald, Germany) and the Laboratory of Pathology and Cytology Bad Berka (Bad Berka, Germany). Several tumour specimens contained neighbouring non-malignant tissue that was also analysed. Furthermore, tumour-free human tissue samples from the cortex, lung, heart, liver, stomach, gut, pancreas, kidney, spleen, thymus, lymph nodes and placenta (*n* = 2–5 each site) were also evaluated. These specimens were obtained from the Department of Pathology of the Ernst Moritz Arndt University (Greifswald, Germany) and the Laboratory of Pathology and Cytology Bad Berka (Bad Berka, Germany).

For the subsequent evaluation of FAM159B expression in bronchopulmonary and gastroenteropancreatic neuroendocrine neoplasms (BP-NEN and GEP-NEN), 764 tumour samples from 278 patients (in detail: 110 × 1, 55 × 2, 44 × 3, 32 × 4, 13 × 5, 8 × 6, 3 × 7, 3 × 8, 3 × 9, 1 × 11, 1 × 12, 3 × 14, 1 × 16 and 1 × 18 samples per patient), with 416 primary tumour samples from 206 patients and 313 metastasis samples from 116 patients, were included in the investigation. For 35 samples from 45 patients, it was not known whether they originated from the primary tumour or the metastasis. Samples from primary tumours and metastases were available for several patients. Of the tumours, 97 (34.9%) originated from the lung (in detail: 24 typical carcinoids (TC), 27 atypical carcinoids (AC), 38 small-cell lung cancer (SCLC) and 8 large-cell neuroendocrine carcinomas (LCNEC)), 18 (6.5%) from the stomach, 16 (5.7%) from the duodenum/jejunum, 58 (20.9%) from the ileum, 5 (1.8%) from the appendix, 11 (4.0%) from the colon, 15 (5.4%) from the rectum and 49 (17.6%) from the pancreas. The localisation of 9 (3.2%) primary tumours was unknown.

The samples were made available by the Institute of Pathology and Cytology Bad Berka (Bad Berka, Germany) and had been surgically removed between 1998 and 2016 at the Department of General and Visceral Surgery, Zentralklinik Bad Berka (Bad Berka, Germany). The clinical data were gathered from the patient records.

All procedures performed in this study involving human participants were in accordance with both the ethical standards of the institutional or national research committee and the 1964 Helsinki declaration and its later amendments. Permission for this retrospective analysis was obtained from the local ethics committee (Ethikkommission der Landesärztekammer Thüringen). All data were recorded and analysed anonymously.

#### 4.4.2. Cell Lines and Cytoblocks

Neuroblastoma cell lines (SiMa, SK-N-SH and SH-SY5Y), a neuroendocrine tumour cell line (BON-1), SCLC cell lines (OH-1, NCI-H69 and NCI-H82), a lung adenocarcinoma cell line (A549), hepatocellular carcinoma cell lines (HepG2, Hep3B, SK-HEP-1 and HuH-7), colon carcinoma cell lines (SW480, HT-29 and LoVo), a renal cell carcinoma cell line (A498), urinary bladder carcinoma cell lines (T24 and RT-112), breast cancer cell lines (MDA-MB-231, MCF-7 and BT-474), ovarian carcinoma cell lines (SK-OV-3 and A2780), cervical cancer cell lines (SW756, SiHa, ME-180, CaSki and HeLa), prostate cancer cell lines (PC-3, DU145 and LNCaP) and an epidermoid carcinoma cell line (A431) (DMSZ, Braunschweig, Germany) were grown in 75 cm^2^ culture flasks to 80% confluence. In preparation for the cytoblocks, the cells were washed once with PBS and transferred into 10% buffered formalin for 2 h. After centrifugation for 10 min at 3500 × *g*, the supernatant was removed, and 1 mL of pooled human plasma was added to the cell samples. The samples were briefly vortexed, 100 µL of fibrinogen was added to each sample and the samples were vortexed again. The resulting clots were immersed in 10% buffered formalin for 24 h and subsequently embedded in paraffin blocks.

#### 4.4.3. Immunohistochemistry

From the paraffin blocks, 4 µm sections were prepared and floated onto positively charged slides. Immunostaining was performed using an indirect peroxidase labelling method as described previously [10]. Briefly, samples were dewaxed and microwaved in 10 mM citric acid (pH 6.0) for 16 min at 600 W and incubated with the anti-FAM159B antibody HPA011778 (dilution 1:100) overnight at 4 °C. The primary antibody was detected via biotinylated anti-rabbit IgG and subsequent incubation of the samples with peroxidase-conjugated avidin (Vector ABC Elite Kit; Vector Laboratories, Burlingame, CA, USA). Bound primary antibody was visualised using 3-amino-9-ethylcarbazole in acetate buffer (BioGenex, San Ramon, CA, USA). Samples were then rinsed and counterstained with Mayer’s haematoxylin and mounted with Vectamount^TM^ mounting medium (Vector Laboratories, Burlingame, CA, USA). For immunohistochemical controls, HPA011778 was adsorbed for 2 h at room temperature with 10 µg/mL of the peptide PrEST antigen (Atlas Antibodies, Bromma, Sweden) used for rabbit immunisations.

For dual immunofluorescence experiments, samples were incubated overnight at 4 °C with anti-FAM159B HPA011778, together with either a mouse monoclonal anti-insulin antibody (dilution 1:100; Abcam, Cambridge, MA, USA) or a mouse monoclonal anti-glucagon antibody (dilution 1:500; Sigma-Aldrich, St. Louis, MO, USA). Next, samples were incubated with Cy3-conjugated anti-rabbit and Alexa-Fluor-488-conjugated anti-mouse antibodies. After mounting (Invitrogen Fluoromount-G, with DAPI; Thermo Fisher Scientific, Waltham, MA, USA) the samples were examined using a Zeiss LSM 510 META laser scanning confocal microscope (Jena, Germany).

FAM159B staining of the tumour slides was scored using the semi-quantitative immunoreactivity score (IRS) according to Remmele and Stegner [11]. The percentage of positive cells was assigned one of five possible gradations (no positive cells (0), <10% positive cells (1), 10–50% positive cells (2), 51–80% positive cells (3) or >80% positive cells (4)) and multiplied by the staining intensity, which could be assigned one of four gradations (no staining (0), mild staining (1), moderate staining (2) or strong staining (3)). The obtained IRS values thus ranged from 0 to 12. Immunohistochemical staining was evaluated by two independent blinded investigators (A.-S.L.B. and A.L.). In the case of discrepancies in scoring, final decisions were achieved by consensus. Because several patients with neuroendocrine tumours had more than one tumour slide, the arithmetic mean was calculated from the IRS of all slides belonging to the same patient. Only tumours with an average IRS ≥ 3 were considered positive for FAM159B. The IRS values representing FAM159B expression were classified as follows: 0–2, negative/no expression; 3–5, low expression; 6–8, moderate expression; 9–12, strong expression. 

#### 4.4.4. Statistics

SPSS 25.0.0.0 (IBM, Armonk, NY, USA) was used for the statistical analyses. Because the data were not normally distributed, a Kolmogorov-Smirnov test, Kruskal–Wallis test, Mann–Whitney test, chi-square test, Kendall’s τ-b test or Spearman’s rank correlation was performed. For survival analysis, the Kaplan–Meier method with a log-rank test was used. *P* values ≤ 0.05 were considered statistically significant.

## 5. Conclusions

We thoroughly characterised the polyclonal rabbit anti-human FAM159B antibody HPA011778 and demonstrated its suitability for both Western blot and immunocytochemistry analyses in basic research, as well as for visualising FAM159B expression in formalin-fixed, paraffin-embedded tissue samples. By applying this antibody, we could detect FAM159B expression in various non-neoplastic and neoplastic tissues and cancer cell lines. Our results also implicate FAM159B in the direct or indirect regulation of diverse membrane proteins, including receptors, ion channels or others such as PD-L1, that may be typically (but not exclusively) expressed in neuronal and (neuro)endocrine tissues. In this context, FAM159B may also regulate secretory functions, among other actions.

## Figures and Tables

**Table 1:** Presence of FAM159B in different tumour entities.

Tumour Type (Total Number of Cases)	FAM159B-Positive Tumours		Immunoreactive Score (IRS)		
	*n*	%	Mean	Min	Max
Glioblastoma (10)	1	10	1.11	0.0	3.0
Pituitary adenoma (2)	1	50	7.00	2.0	12.0
Thyroid carcinoma (37)	29	78.4	4.90	0.0	12.0
- papillary (10)	9	90	4.40	2.0	6.0
- follicular (10)	4	40	2.40	0.0	6.0
- medullary (9)	9	100	6.90	3.0	12.0
- anaplastic (8)	7	87.5	6.40	1.5	12.0
Parathyroid adenoma (10)	9	90	5.10	2.0	8.0
Lung cancer (30)	26	86.7	5.18	1.0	12.0
- Adenocarcinoma (10)	10	100	5.60	3.0	12.0
- Squamous cell carcinoma (10)	6	60	4.30	1.0	9.0
- Small-cell lung cancer (10)	10	100	6.25	3.0	10.0
Gastric adenocarcinoma (10)	6	60	4.40	0.0	9.0
Colon carcinoma (9)	6	66.7	3.50	0.0	6.0
Gastrointestinal stromal tumour (10)	2	20	1.95	0.0	9.0
Hepatocellular carcinoma (11)	3	27.3	1.36	0.0	4.0
Cholangiocellular carcinoma (8)	3	37.5	2.63	0.0	9.0
Pancreatic adenocarcinoma (10)	6	60	3.25	0.0	6.0
Renal clear cell carcinoma (10)	1	10	1.50	0.0	3.0
Urinary bladder carcinoma (7)	2	28.6	2.00	0.0	4.0
Pheochromocytoma (7)	3	42.9	2.29	0.0	4.0
Breast carcinoma (9)	5	55.6	3.17	0.0	6.0
Ovarian cancer (9)	8	88.9	5.39	2.0	9.0
Endometrial cancer (10)	5	50	2.50	0.0	9.0
Cervical cancer (9)	5	55.6	4.00	0.0	12.0
Prostate adenocarcinoma (12)	4	33.3	2.17	0.0	6.0
Testicular cancer (10)	2	20	2.10	0.0	6.0
Lymphoma (10)	9	90	6.40	0.0	10.0
Melanoma (5)	3	60	4.00	2.0	6.0
Sarcoma (14)	1	7.1	0.71	0.0	6.0
- Liposarcoma (4)	0	0	0.00	0.0	0.0
- Rhabdomyosarcoma (4)	1	25	2.50	0.0	6.0
- Leiomyosarcoma (4)	0	0	0.00	0.0	0.0
- Pleomorphic sarcoma (2)	0	0	0.00	0.0	0.0

**Table 2:** Correlations of FAM159B IRS values with neuroendocrine tumour markers.

Correlation between FAM159B Expression and:		Correlation-Coefficient (r_sp_)	Level of Significance (*p*)
**Ki-67 index**	All tumours	0.306	<0.001
	GEP-NEN	0.279	<0.001
	BP-NEN	0.218	<0.001
**IRS CgA**	All tumours	0.037	0.337
	GEP-NEN	0.001	0.983
	BP-NEN	0.096	0.122
**IRS D2-receptor**	All tumours	0.215	<0.001
	GEP-NEN	0.281	<0.001
	BP-NEN	0.041	0.542
**IRS SST1**	All tumours	0.156	<0.001
	GEP-NEN	0.166	0.001
	BP-NEN	−0.117	0.056
**IRS SST2**	All tumours	0.042	0.267
	GEP-NEN	0.177	<0.001
	BP-NEN	−0.049	0.421
**IRS SST3**	All tumours	0.154	<0.001
	GEP-NEN	0.216	<0.001
	BP-NEN	−0.024	0.699
**IRS SST4**	All tumours	0.155	<0.001
	GEP-NEN	0.076	0.117
	BP-NEN	0.151	0.013
**IRS SST5**	All tumours	0.234	<0.001
	GEP-NEN	0.313	<0.001
	BP-NEN	−0.048	0.438
**IRS CXCR4**	All tumours	0.253	<0.001
	GEP-NEN	0.234	<0.001
	BP-NEN	0.173	0.006
**IRS PD-L1**	All tumours	0.440	<0.001
	GEP-NEN	0.467	<0.001
	BP-NEN	0.201	0.056

BP-NEN: bronchopulmonary neuroendocrine neoplasms; CXCR4: chemokine receptor 4; D2: dopamine receptor; GEP-NEN: gastroenteropancreatic neuroendocrine neoplasms; PD-L1: programmed death ligand 1; SST: somatostatin receptor.

## Data Availability

The data that support the findings of this study are all contained within the article.

## Abbreviations

AT: atypical carcinoids; BP-NEN: bronchopulmonary neuroendocrine neoplasms; GEP-NEN: gastroenteropancreatic neuroendocrine neoplasms; IRS: immunoreactivity score; PD-L1: programmed death ligand 1; PBS: phosphate-buffered saline; SCLC: small-cell lung cancer; SST: somatostatin receptor; TC: typical carcinoids; WGA: wheat germ agarose.
